# Phase 1 and dose-finding study of patritumab (U3-1287), a human monoclonal antibody targeting HER3, in Japanese patients with advanced solid tumors

**DOI:** 10.1007/s00280-014-2375-2

**Published:** 2014-01-18

**Authors:** Hiroshi Wakui, Noboru Yamamoto, Shinji Nakamichi, Yousuke Tamura, Hiroshi Nokihara, Yasuhide Yamada, Tomohide Tamura

**Affiliations:** 1 Department of Thoracic Oncology, National Cancer Center Hospital, 5-1-1, Tsukiji, Chuo-ku, Tokyo, 104-0045 Japan; 2 Department of Gastrointestinal Oncology, National Cancer Center Hospital, 5-1-1, Tsukiji, Chuo-ku, Tokyo, 104-0045 Japan

**Keywords:** U3-1287, Patritumab, HER3, Phase 1 study, Solid tumor

## Abstract

**Purpose:**

Patritumab (U3-1287) is a human epidermal growth factor receptor-3 (HER3)-targeted antibody that blocks ligand-associated activation of HER3. This open-label, phase 1 and dose-finding study (ClinicalTrials.jp Identifier: JapicCTI-101262) aimed to assess the safety, pharmacokinetics, incidence of anti-patritumab antibody, recommended dose for subsequent clinical studies, preliminary efficacy, and patritumab-related biomarkers in Japanese patients with advanced solid tumors.

**Methods:**

Patients received patritumab 9 or 18 mg/kg intravenously every 3 weeks until disease progression or intolerable toxicity occurred. Adverse events (AEs) were assessed according to the National Cancer Institute Common Terminology Criteria for Adverse Events (CTCAE version 4.0). Dose-limiting toxicities (DLTs) were evaluated from the initial dose to Cycle 1 Day 21. Tumor response was assessed with Response Evaluation Criteria in Solid Tumors (RECIST version 1.1).

**Results:**

Nine patients received patritumab 9 mg/kg (*n* = 3) or 18 mg/kg (*n* = 6). Five patients were male, all patients had Eastern Cooperative Oncology Group performance status (PS) ≤ 1, and median (range) age of 67 (50–69) years. No DLTs were reported. Patritumab-related AEs reported in ≥2 patients were ALT increase (three patients), thrombocytopenia, diarrhea, stomatitis, cheilitis, rash maculo-papular and AST increase (two each). Pharmacokinetics profile was similar to the preceding US phase 1 study. Soluble HER3 concentration in serum unexpectedly increased in all patients. These changes did not correlate with clinical response. Four patients had a best response of stable disease. All patients tested had negative for anti-patritumab antibody formation.

**Conclusions:**

Patritumab was well tolerated up to 18 mg/kg without DLTs in Japanese patients with advanced solid tumors. Soluble HER3 increased in all patients.

## Introduction

The human epidermal growth factor receptor-3 (HER3) is expressed in many normal tissues and in a variety of solid tumors [1, 2]. Unlike other HER family members, HER3 lacks intrinsic kinase activity [3]. However, the cytoplasmic tail of HER3 contains six docking sites for the p85 regulatory subunit of phosphatidylinositol-3-kinase (PI3K) and serves as a scaffold for PI3K/protein kinase B signaling for the HER family via heterodimeric interactions with other HER family members [4–6]. Increased levels of HER3 have been associated with a negative clinical prognosis, including survival in several tumor types [7–11]. Recent data suggest that HER3 is involved in resistance to other HER receptor-targeted therapeutics, e.g., trastuzumab, lapatinib, cetuximab, gefitinib and erlotinib [12–16].

Patritumab (U3-1287) is a fully human monoclonal immunoglobulin G1 (IgG1) antibody directed against HER3. Patritumab inhibits ligand binding (heregulin alpha and heregulin beta) and receptor activation and induces HER3 down-regulation. Functionally, patritumab inhibits tumor cell proliferation, survival and anchorage-independent growth in vitro, and inhibits growth of HER3 expressing xenograft tumor models in vivo [17–20].

In a preceding US Phase 1 study (ClinicalTrials.gov Identifier: NCT00730470) [21], the tolerability of patritumab was evaluated up to the dose of 20 mg/kg without dose-limiting toxicities (DLTs). The most frequently reported patritumab-related adverse events (AEs) were fatigue (12/57, 21.1 %), diarrhea (7/57, 12.3 %) and nausea (6/57, 10.5 %). The majority of patritumab-related AEs were Grade 1 or 2. This was an open-label, phase 1 and dose-finding study to evaluate the safety, pharmacokinetics (PK) of repeated administration of patritumab and preliminary efficacy in Japanese patients with advanced solid tumors.

## Patients and methods

### Patient eligibility

Eligible patients had histologically or cytologically confirmed advanced solid tumors that were refractory to standard treatment and were well known to express HER3 (e.g., lung, breast, colorectal, cervical, esophageal and sarcoma). Eligibility criteria also included the following: age 20–75 years at informed consent; Eastern Cooperative Oncology Group (ECOG) performance status (PS) 0–1; life expectancy greater than 3 months; no previous chemotherapy, radiation therapy, hormonal therapy or surgery within 4 weeks before treatment with patritumab (6 weeks for previous treatment with nitrosoureas or mitomycin C); and adequate hematologic, hepatic and renal function. Any toxicity related to prior therapy must have recovered. Exclusion criteria included: previous treatment with other anti-HER3-targeted therapy; symptomatic brain metastasis; pleural effusion and ascites that required drainage; a history of thromboembolic disease or bleeding diatheses; serious preexisting medical conditions such as uncontrolled infections, severe cardiovascular or cerebrovascular disease, uncontrolled hypertension or diabetes mellitus, chronic diarrhea, inflammatory bowel disease, partial ileus and psychiatric disorders; a history of hypersensitivity reactions to any drugs; a history of serious hypersensitivity to drug containing polysorbate 20; hepatic B or C virus or human immunodeficiency virus infection; pregnancy or lactation; and not willing to use contraception during and after 6 months of the study.

Written informed consent was obtained from all patients. This study was conducted in accordance with the Declaration of Helsinki and the applicable guidelines on good clinical practice, and the protocol and the informed consent received institutional review board/independent ethics committee approval.

### Study design

This was an open-label, phase 1 and dose-finding study conducted at National Cancer Center Hospital, Tokyo, Japan. The primary objective was to evaluate the safety and PK of patritumab in Japanese patients with advanced solid tumors. Secondary objectives were to determine recommended dose for subsequent clinical studies, to evaluate incidence of anti-patritumab antibody, to preliminary assess tumor response and to explore patritumab-related biomarkers.

### Drug administration and dose-escalation procedure

Eligible patients received intravenous administration of patritumab at a dose of 9 or 18 mg/kg every 3 weeks until disease progression or intolerable toxicity occurred.

DLTs were evaluated from the initial dose to Cycle 1 Day 21. DLTs were defined as any of the following toxicities assessed to have a causal relationship with the study drug: febrile neutropenia (≥38.5 °C, neutrophil count <1,000/μL) or Grade 4 neutropenia persisting for more than 7 days; Grade 4 thrombocytopenia or Grade 3 thrombocytopenia requiring blood transfusion; uncontrollable Grade 3 or worse fatigue, anorexia, nausea, vomiting or diarrhea despite maximal supportive treatment; and Grade 3 or worse toxicities except for above three definitions. However, fever without neutropenia and transient electrolyte abnormality did not qualify as DLTs.

Dose levels were escalated from 9 mg/kg according to the typical 3 + 3 design.

### Safety assessments

AEs were evaluated according to the National Cancer Institute Common Terminology Criteria for Adverse Events (CTCAE version 4.0) throughout the treatment period until 30 days after the last dose. Safety evaluations were based upon medical review of AEs and the results of clinical laboratory tests, vital sign measurements, 12-lead electrocardiograms, physical examination, ECOG PS, X-ray/computed tomography scan. Anti-patritumab antibody was assessed before each treatment cycle. Anti-patritumab antibody was measured by Electrochemiluminescence Immunoassay.

### Pharmacokinetic analyses

Blood samples for PK evaluation were collected on pre-dose and 0.25, 4, 7, 24 and 72 h after the first dose, on Day 8 and 15 of Cycle 1, Day 1 of Cycle 2, 3, and 4 and Day 30 of last Cycle. The U3-1287 level was measured by enzyme-linked immunosorbent assay (ELISA). PK parameters [AUC_0–21 days_, *C*_max_, CL, *V*_ss_, and terminal half-life] were derived from serum patritumab concentration versus time. The PK parameters for the first dose were calculated by a non-compartmental analysis using the computer software WinNonlin (Ver5.2, Pharsight Corp., CA, USA). PK statistical analyses were performed by SAS System Release 9.1.3 (SAS Institute Japan Ltd., Tokyo, Japan).

### Tumor response

Tumor response was determined for all patients with measurable and/or non-measurable lesions according to Response Evaluation Criteria in Solid Tumors (RECIST version 1.1). Tumor measurements by CT or MRI were obtained at baseline, every 6 weeks thereafter and Cycle 1 Day 21.

### Serum HER3

Blood was collected on Day 1 (before administration), 2, 8 and 15 of Cycle 1, Day 1 (before administration) of even-numbered cycles, and during the follow-up period to evaluate the change in the serum soluble HER3 level. The soluble HER3 level was measured by ELISA.

### Biomarkers using tumor tissue

Biomarker research using the tumor tissue was performed for only patients who had written informed consent to participate in biomarker research aside from the study. Paraffin-embedded samples of tumor tissue archived before enrollment (whenever it was collected) were used to evaluate the items below at Mosaic Laboratories.

- HER3 protein expression level (by IHC method)
- Frequency of HER3 gene amplification (by FISH method)

### Statistical method

All patients who received study medication were included in the analysis of safety and efficacy. Safety and efficacy statistical analyses were performed by SAS System Release 8.2 (SAS Institute Japan Ltd., Tokyo, Japan).

## Results

### Patient characteristics

Nine patients were enrolled and treated in this study; three and six patients received patritumab 9 and 18 mg/kg administration, respectively. Patient characteristics are listed in Table 1. There were 5 males with a median age of 67 (range 50–69) years. Tumor types included 2 non-small-cell lung cancer (NSCLC), 2 esophageal cancer, 2 colorectal cancer, 1 breast cancer, 1 cervical cancer and 1 sarcoma. All patients had received previous treatment for their cancer. The median number of prior chemotherapy regimens (range) was 4 (2–7).

**Table 1:** Patient characteristics

	9 mg/kg (%)	18 mg/kg (%)	Total (%)
All enrolled	3	6	9
Gender			
Male	2	3	5
Female	1	3	4
ECOG PS			
0	1 (33.3)	2 (33.3)	3 (33.3)
1	2 (66.7)	4 (66.7)	6 (66.7)
Age (year)			
Median (range)	63 (50–69)	67.5 (51–69)	67 (50–69)
Primary tumor type			
NSCLC	2 (66.7)	0	2 (22.2)
Esophageal cancer	1 (33.3)	1 (16.7)	2 (22.2)
Colorectal cancer	0	2 (33.3)	2 (22.2)
Breast cancer	0	1 (16.7)	1 (11.1)
Cervical cancer	0	1 (16.7)	1 (11.1)
Sarcoma	0	1 (16.7)	1 (11.1)
Prior chemotherapy regimens			
Median (range)	3 (2–4)	5 (2–7)	4 (2–7)

### Safety and tolerability

No DLTs were reported in this study. The patritumab-related AEs reported in ≥2 patients were ALT increased (*n* = 3), thrombocytopenia (*n* = 2), diarrhea (*n* = 2), stomatitis (*n* = 2), cheilitis (*n* = 2), rash maculo-papular (*n* = 2) and AST increased (*n* = 2) (Table 2).

**Table 2:** Number of patients with major treatment-related AEs in all cycles

	9 mg/kg (*N* = 3)				18 mg/kg (*N* = 6)				Total (*N* = 9)
	Grade 1	Grade 2	Grade 3	Any grades (%)	Grade 1	Grade 2	Grade 3	Any grades (%)	Any grades (%)
*Gastrointestinal disorders*									
Cheilitis	1	0	0	1 (33.3)	1	0	0	1 (16.7)	2 (22.2)
Diarrhea	0	0	0	0	2	0	0	2 (33.3)	2 (22.2)
Stomatitis	0	0	0	0	2	0	0	2 (33.3)	2 (22.2)
*Skin and subcutaneous tissue disorders*									
Rash maculo-papular	1	1	0	2 (66.7)	0	0	0	0	2 (22.2)
*Blood examination*									
ALT increased	2	0	0	2 (66.7)	0	1	0	1 (16.7)	3 (33.3)
AST increased	1	0	0	1 (33.3)	1	0	0	1 (16.7)	2 (22.2)
Thrombocytopenia	0	0	0	0	2	0	0	2 (33.3)	2 (22.2)

The majority of patritumab-related AEs were Grade 1 or 2 with the exception of Grade 3 lymphopenia on Cycle1 and Grade 3 interstitial lung disease (ILD) and bacterial pneumonia on Cycle 3 observed in 1 patient. No Grade 4 or 5 AEs were reported throughout the study. Grade 3 lymphopenia occurred during the DLT evaluation period and was assessed as related to the study drug but resolved without intervention, and no significant changes in neutrophil count were observed. One patient in 18 mg/kg experienced three serious adverse events (SAEs): ILD, bacterial pneumonia and ascites. Two of them (ILD and bacterial pneumonia) assessed as related to the study drug were recovered. No AEs leading to treatment discontinuation or death were reported in this study. No notable changes were found in vital signs, 12-lead ECG, body weight or ECOG PS. All patients tested had negative for anti-patritumab antibody.

### Pharmacokinetics

**Fig. 1:**
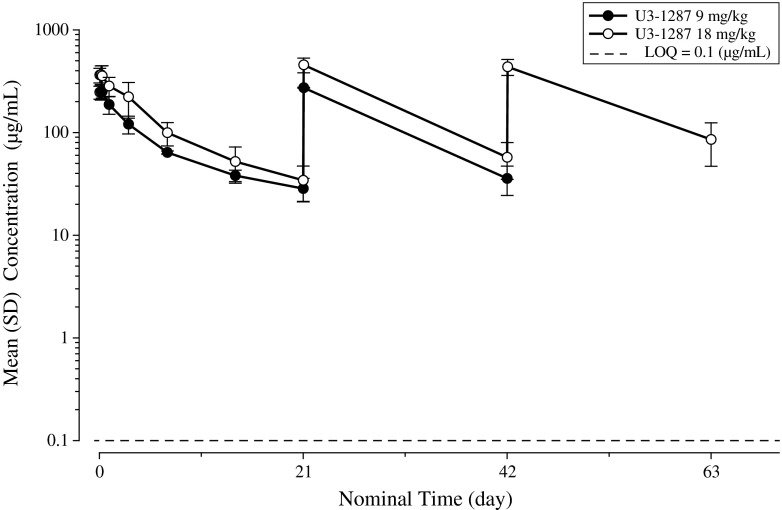
Plasma concentration–time profiles of patritumab from baseline to 3 week of exposure

**Fig. 2:**
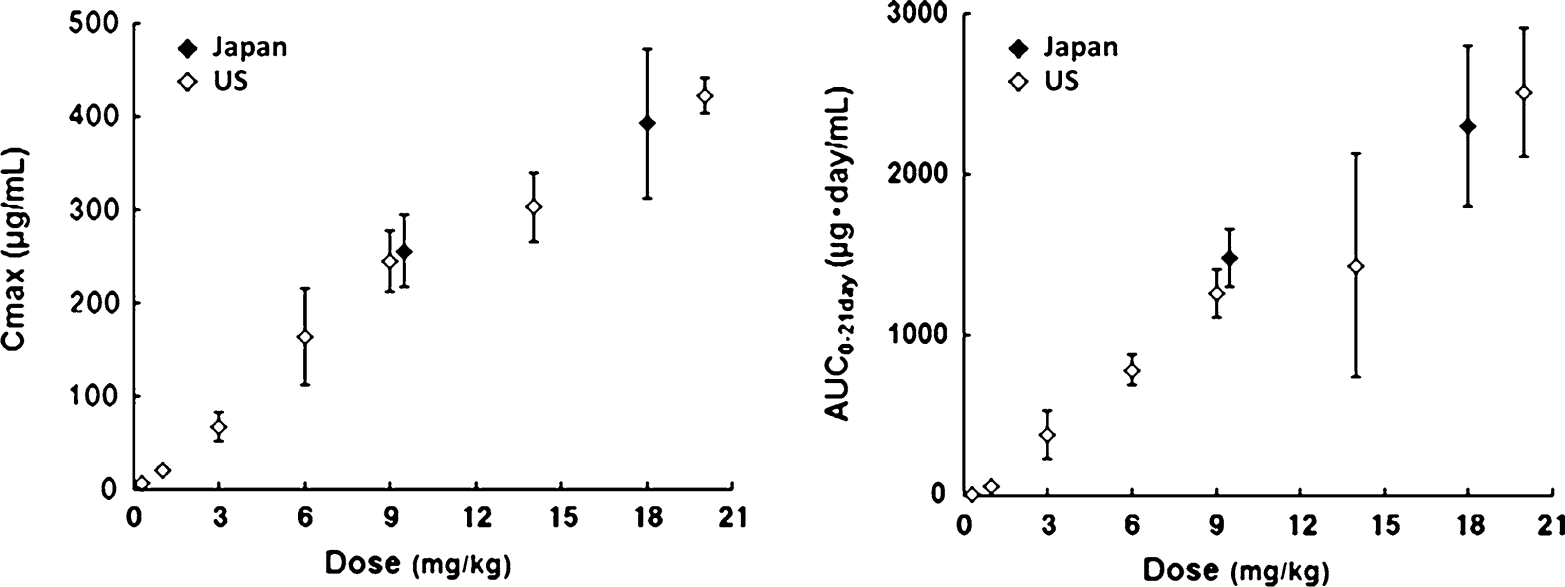
Pharmacokinetics parameters in Phase 1 studies in Japan and US

The PK parameters of patritumab are summarized in Table 3. Plasma disappearance was biphasic and terminal half-life at the dose of 9 and 18 mg/kg was 10.2 and 8.98 days, respectively. No accumulation due to repeated administration was observed (Fig. 1). PK profile of patritumab in this study was similar to that in the US phase 1 study (Fig. 2).

**Table 3:** Pharmacokinetic parameters of patritumab

Pharmacokinetic parameters	9 mg/kg (*N* = 3)	18 mg/kg (*N* = 6)
	mean ± SD	mean ± SD
AUC_0–21day_ (μg day/mL)	1,480 ± 181	2,300 ± 505
*C*_max_ (μg/mL)	255 ± 39	392 ± 80
CL (mL/day/kg)	4.78 ± 0.54	6.85 ± 1.65
*V*_**ss**_ (mL/kg)	61.6 ± 10.0	71.9 ± 23.8
*t*_**1/2**_ (day)	10.2 ± 0.4	8.98 ± 1.62

### Tumor response

Among the 9 patients evaluated in this study, 4 patients had stable disease (SD): NSCLC (1), colorectal cancer (1), breast cancer (1) and sarcoma (1); 3 patients had progressive disease (PD); and 2 were not evaluated. Duration of SD was approximately 16 weeks for NSCLC and colorectal cancer patients, and approximately 10 weeks for breast cancer and sarcoma patients.

### Soluble HER3

**Fig. 3:**
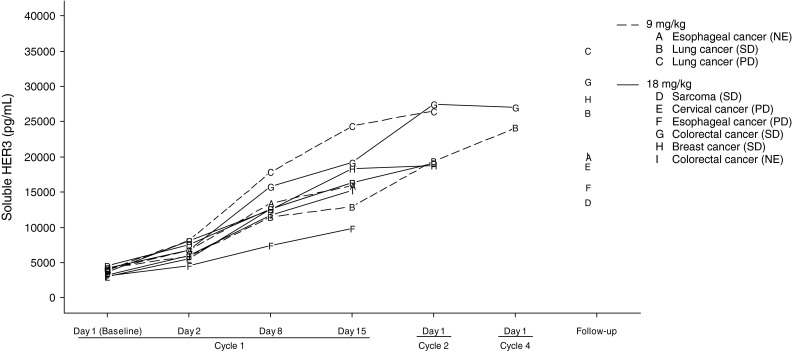
Soluble HER3 concentration in serum, *A*–*I* represent individual patients followed by tumor types and best response inside parenthesis

Soluble HER3 (sHER3) concentration in serum increased in all patients after administration of patritumab (Fig. 3). Mean sHER3 concentration on Cycle 1 Day 1 (baseline) and Cycle 2 Day 1 was 4,086.00 pg/mL and 22,988.50 pg/mL at 9 mg/kg, and 3,646.08 and 21,774.50 pg/mL at 18 mg/kg, respectively. These changes did not correlate with clinical response in 9 patients.

### Biomarkers using tumor tissue

Tumor tissues for biomarker research were provided by two patients. One was esophageal cancer and the other was colorectal cancer, both received patritumab 18 mg/kg, whose best response was PD and NE, respectively. For esophageal cancer patient, no HER3 was observed on cell membrane, ratio of HER3/CEN12 was 1.03 and average signal copy number of HER3 was 3.30. For colorectal cancer patient, cell membrane HER3 level was 90 % of class 0, 5 % of class 1+ and 5 % of class 2+, ratio of HER3/CEN12 was 1.22 and average signal HER3 was 3.90.

## Discussion

This phase 1 and dose-finding study was conducted primarily to evaluate the safety after repeated administration of patritumab in Japanese patients. The safety, PK, anti-patritumab antibody, recommended dose for subsequent clinical studies, tumor response of patritumab, sHER3 and biomarkers were explored in this study.

The target *C*_trough_ was 15 μg/mL which was expected to sufficiently inhibit HER3 activation in humans based on the results of nonclinical PK/PD analyses. Only initial *C*_trough_ before administration in Cycle 2 was lower than 15 μg/mL at the dose of 9 mg/kg every 3 weeks, and all *C*_trough_ in every Cycles were higher than the twice of the target *C*_trough_ at the dose of 18 mg/kg every 3 weeks by PK/PD simulation of the US phase 1 study [21]. Therefore, we determined two dose levels (9 and 18 mg/kg) in this study.

As to safety, no DLTs were reported at any dose level, and the tested doses did not reach the MTD. In the preceding US phase 1 study, patritumab-related AEs were reported in 26 patients (45.6 %) including fatigue (21.1 %), diarrhea (12.3 %), nausea (10.5 %), decreased appetite (7.0 %) and dysgeusia (5.3 %). Like US phase 1 study, toxicities in this study were mild to moderate and were manageable. No infusion reaction during or after patritumab administration was reported in this study as reported in the US phase 1 study. One patient in 18 mg/kg developed bacterial pneumonia and interstitial pneumonia with consolidation and bilateral ground-glass opacity by CT scans on Cycle 3. The patient received antibiotics and steroid therapy, and immediately recovered. It was unclear that interstitial pneumonia was induced by patritumab.

The mean serum patritumab concentration increased as patritumab dose increased. The mean serum patritumab concentration before administration in Cycle 2 was 28.5 μg/mL at level 1 (9 mg/kg) and 34.2 μg/mL at level 2 (18 mg/kg), which were both higher than the target *C*_trough_ of 15 μg/mL. There seems to be similarity between PK profiles of patritumab in this study and in the US phase 1 study.

sHER3 increased over time after patritumab administration in all patients. Several reports described that serum soluble HER2 (sHER2) level increases with tumor progression [22, 23]; however, the level of serum sHER3 increased in all patients independently from best responses. The mechanism of sHER3 increment remains unclear; therefore, further investigation is warranted.

In summary, Patritumab was well tolerated up to 18 mg/kg without DLTs in Japanese patients with advanced solid tumors. PK profiles in Japanese patients were similar to the US phase 1 study. Soluble HER3 increased in all patients after patritumab administration.
